# Supplementary figures and images for: P16 methylation increases the sensitivity of cancer cells to the CDK4/6 inhibitor palbociclib
Source: PLoS One. 2019 Oct 25;14(10):e0223084. doi: 10.1371/journal.pone.0223084 (PMC6814222; doi:10.1371/journal.pone.0223084)

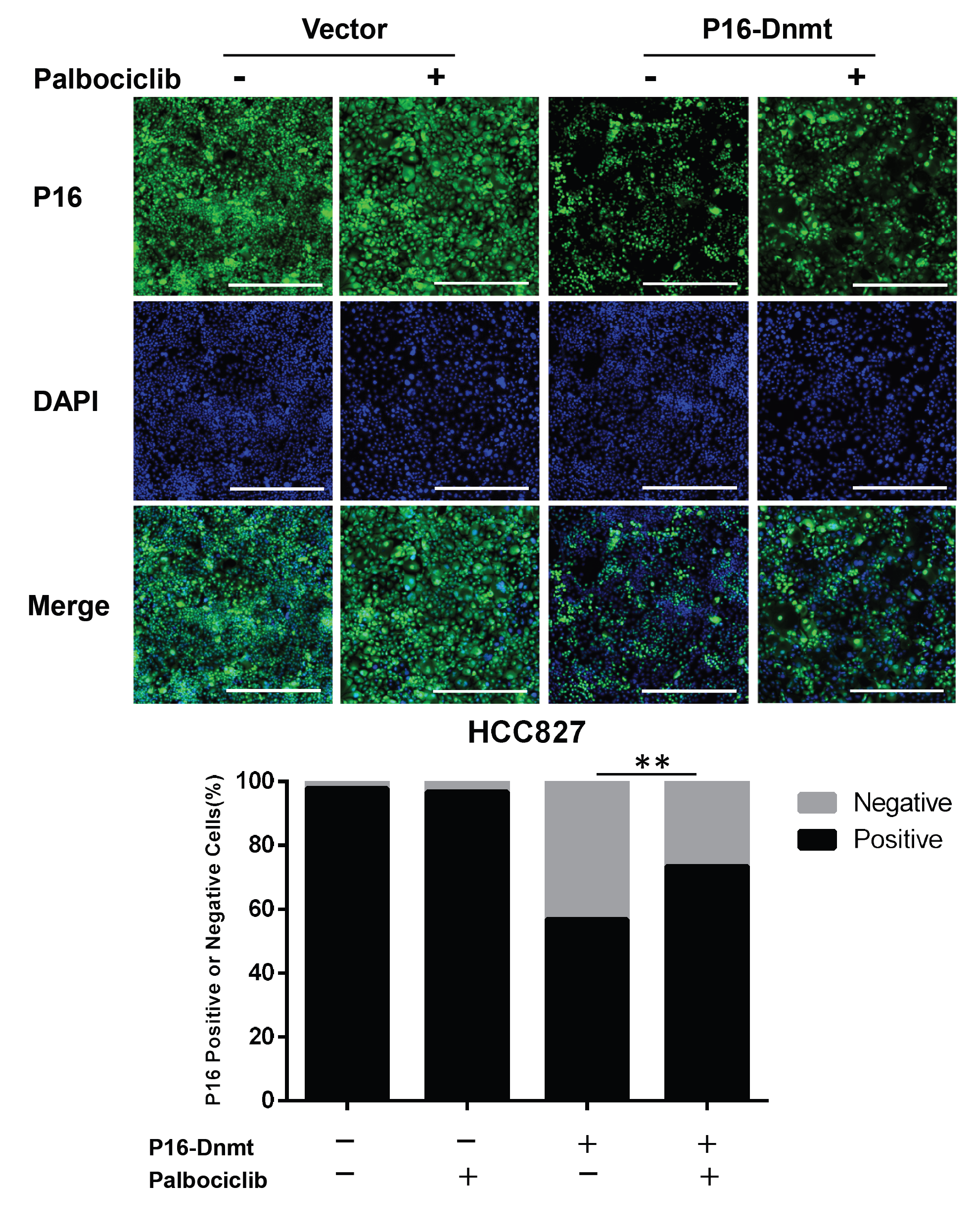

Supplement: S1 Fig — The results of immunofluorescence and confocal microscopy analyses to directly detect alterations in the subpopulation of cells staining positive for P16 protein within HCC827 lung cancer cells stably transfected with P16-Dnmt. Scale bar, 400 μm. Student’s t-test, **p<0.01. (TIF) [file pone.0223084.s001.tif]

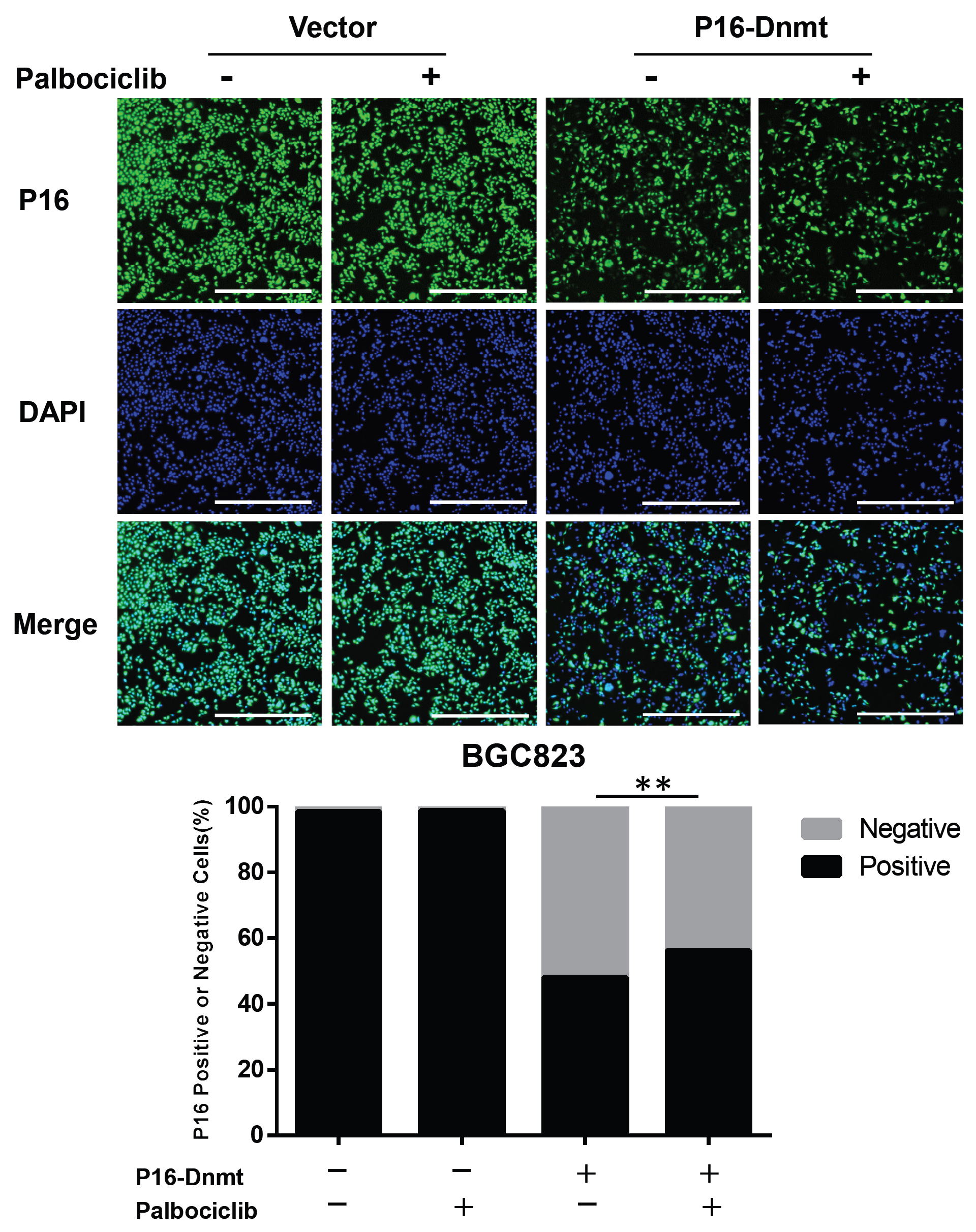

Supplement: S2 Fig — The results of immunofluorescence and confocal microscopy analysis to directly detect alterations of the subpopulation of cells staining positive for P16 protein within the BGC823 gastric cancer cells stably transfected with P16-Dnmt. Scale bar, 400 μm. Student’s t-test, **p<0.01. (TIF) [file pone.0223084.s002.tif]
