# Supplementary material for: P16 methylation increases the sensitivity of cancer cells to the CDK4/6 inhibitor palbociclib
Source: PLoS One. 2019 Oct 25;14(10):e0223084. doi: 10.1371/journal.pone.0223084 (PMC6814222; doi:10.1371/journal.pone.0223084)

Figure 1c

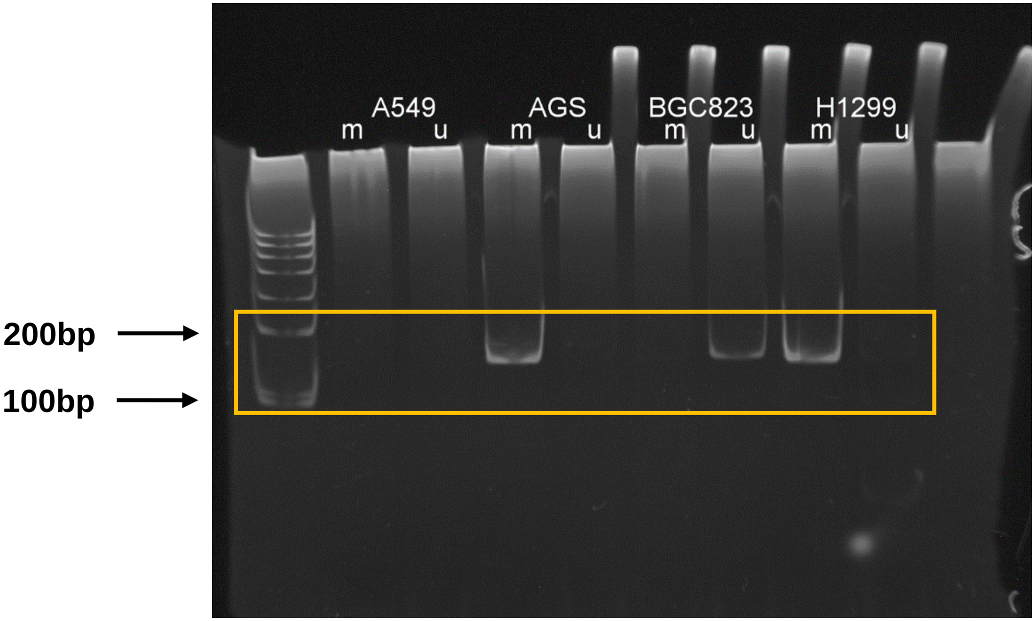

Figure 1c

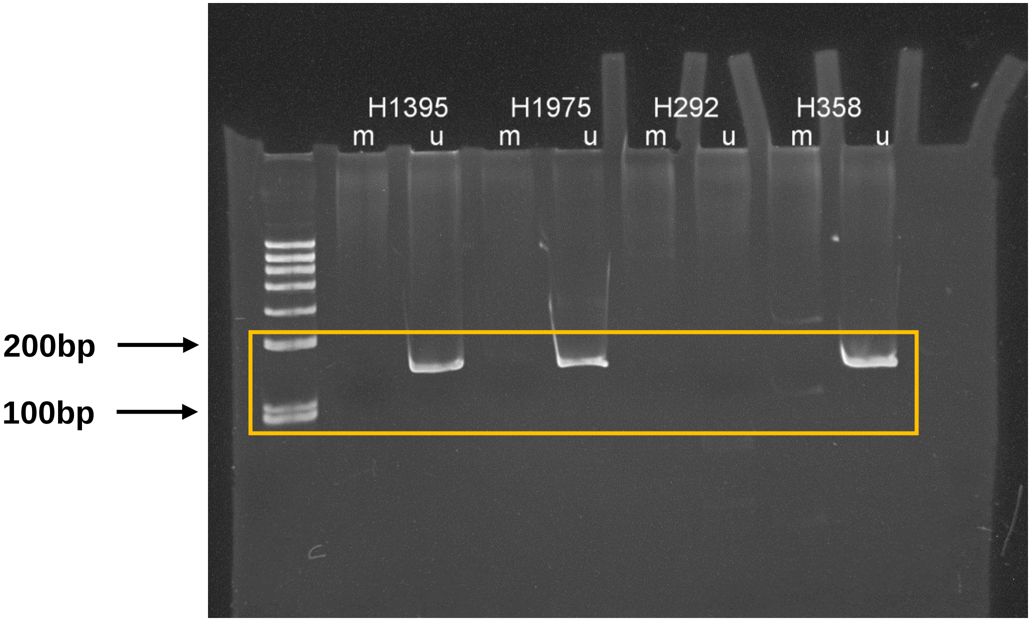

Figure 1c

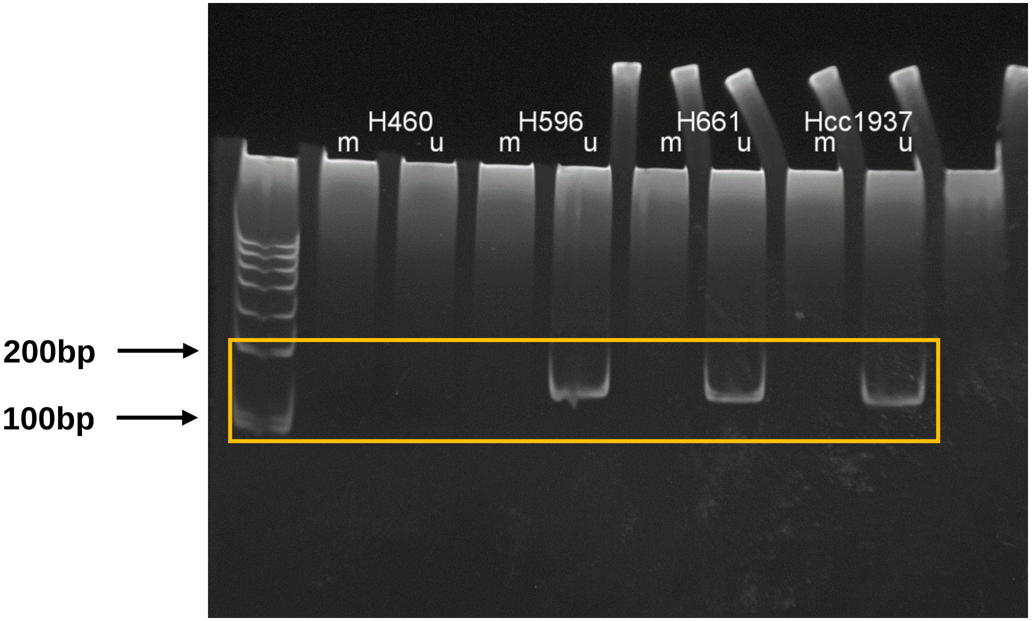

Figure 1c

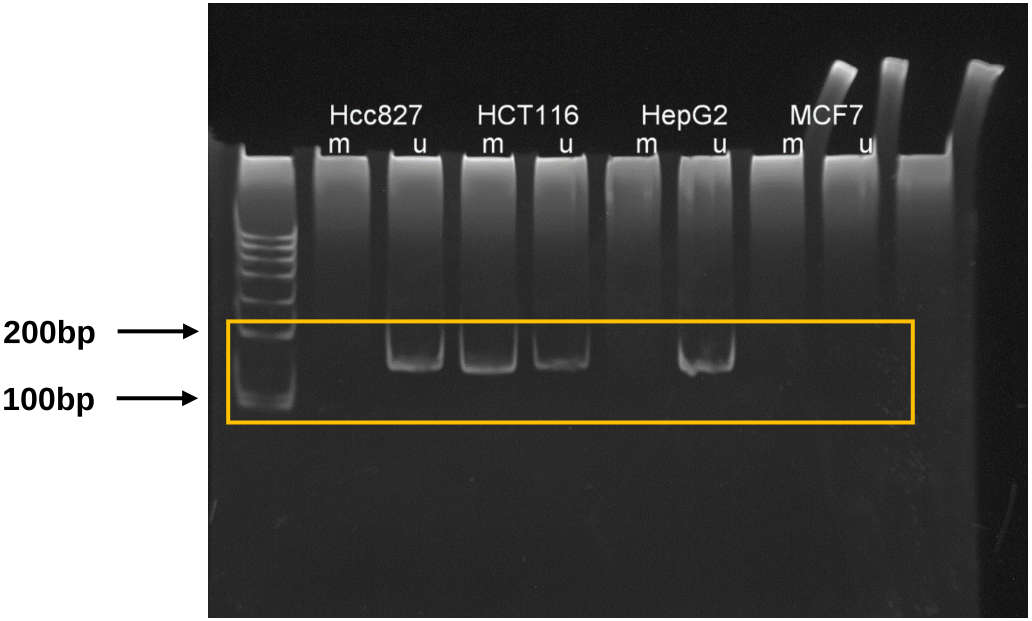

Figure 1c

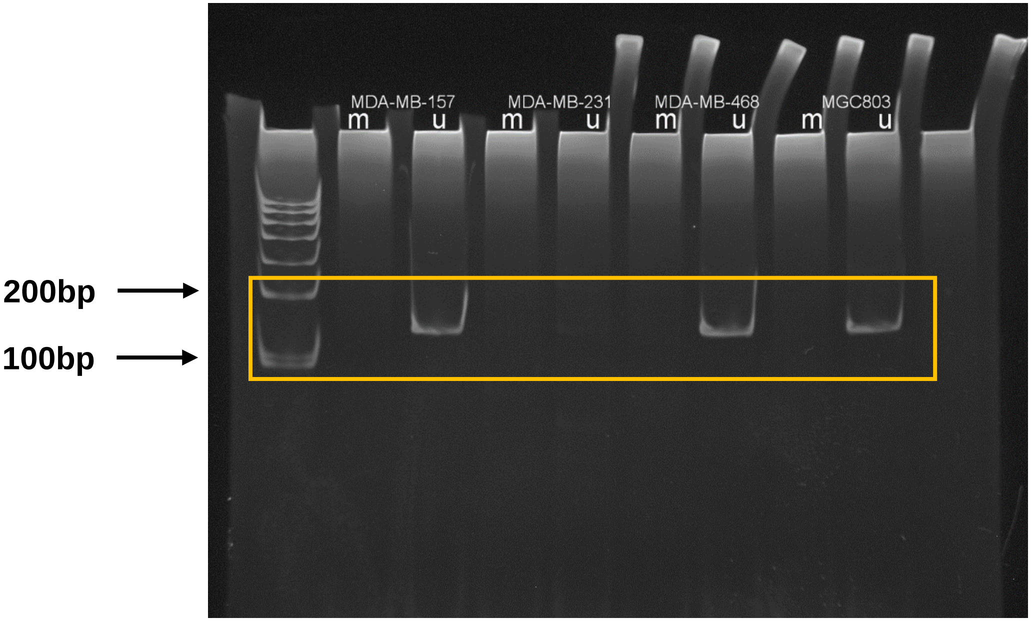

Figure 1c

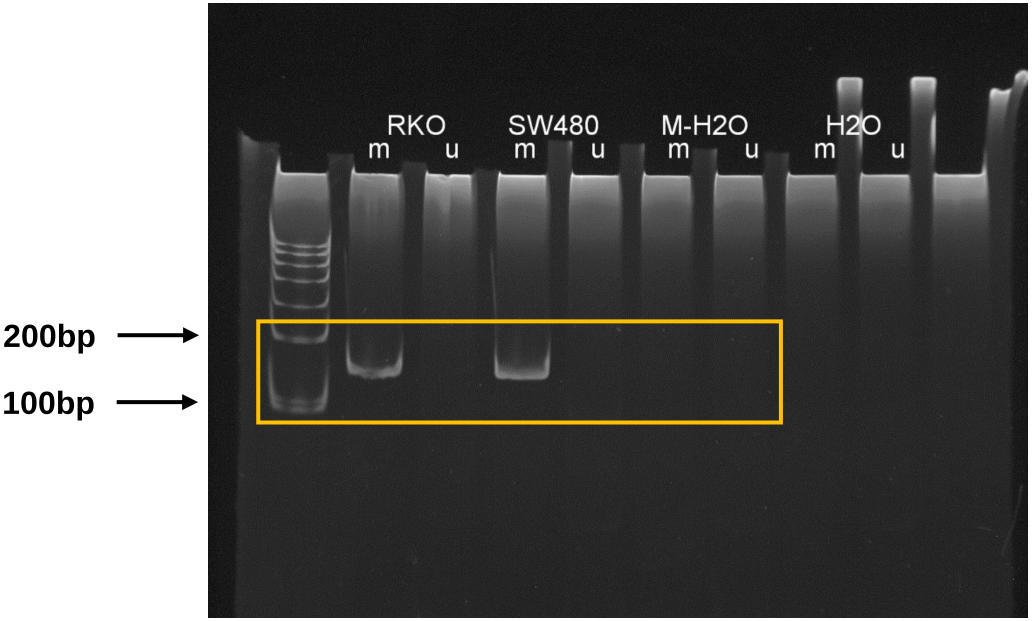

Figure 2a

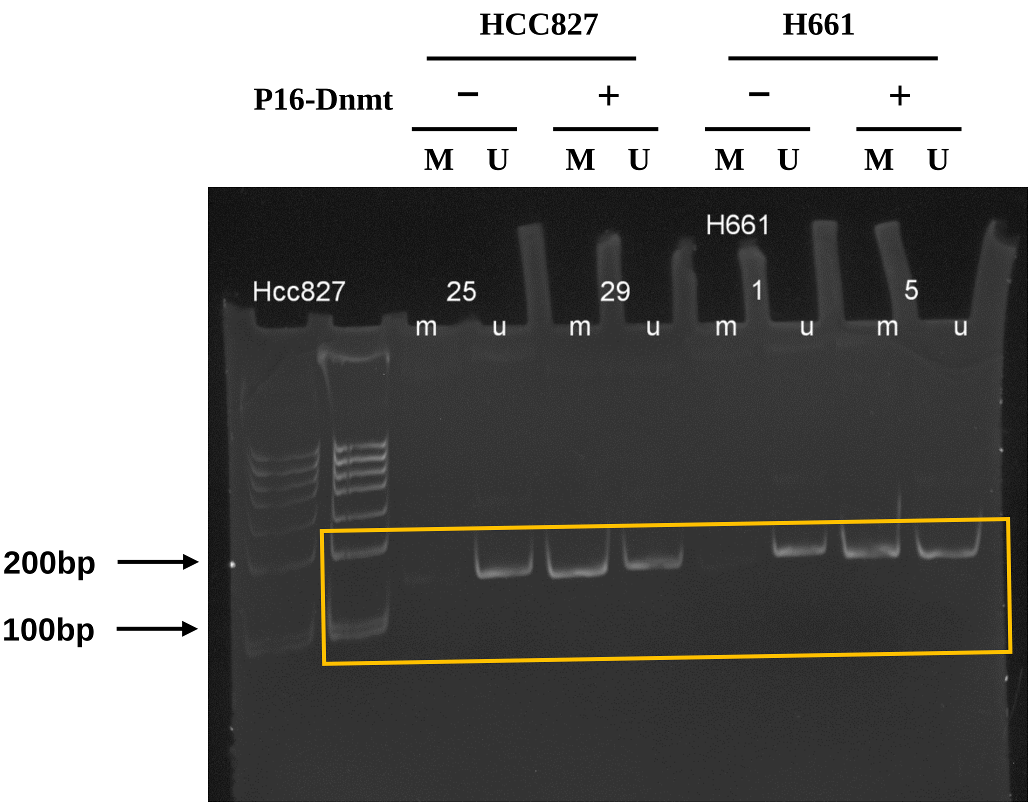
HCC827
H661
P16-Dnmt
−
+
−
+
M U M U M U M U
H661
Hcc827
25
29
1
5
m u m u m u m u
200bp
100bp

Figure 2a

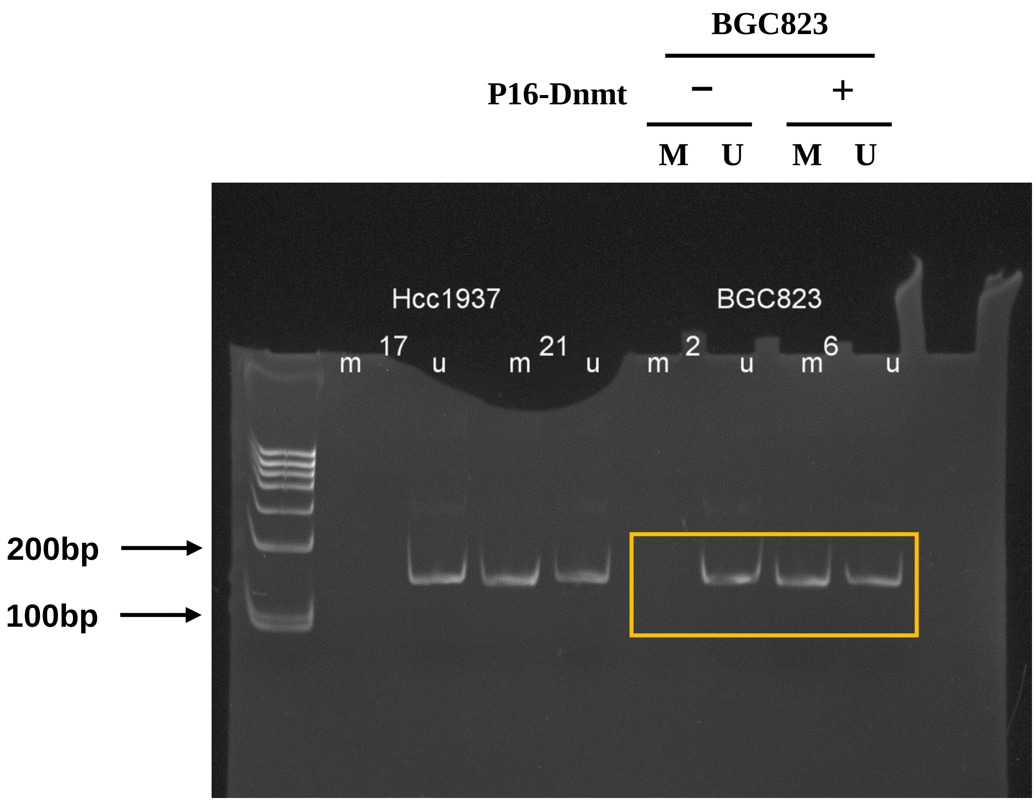
BGC823
P16-Dnmt
−
+
M U M U
Hcc1937
BGC823
m 17 u m 21 u m 2 u m 6 u
200bp
100bp

Figure 2a

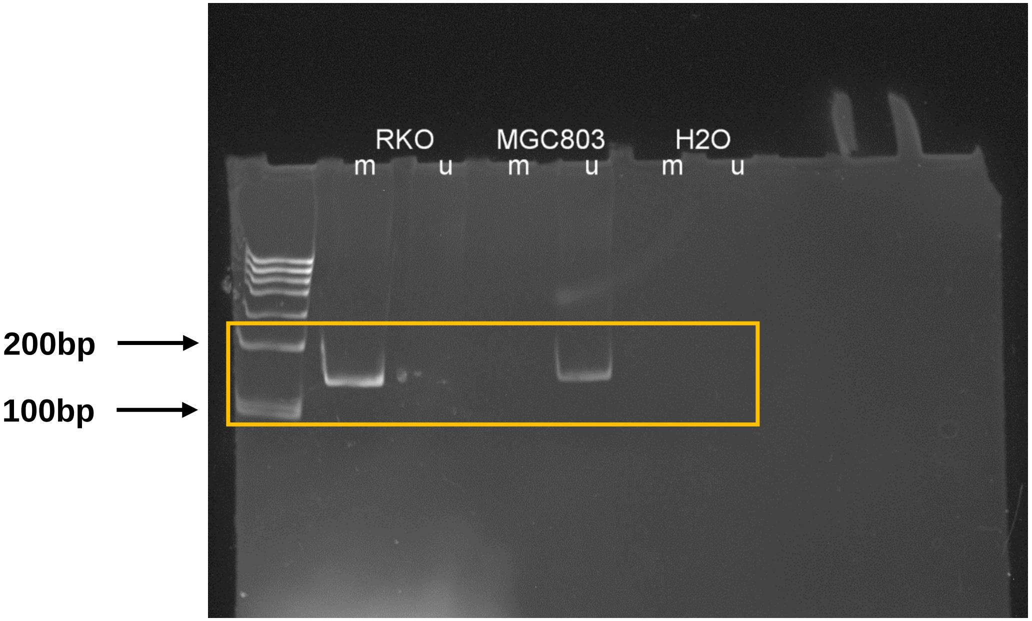

Figure 4d

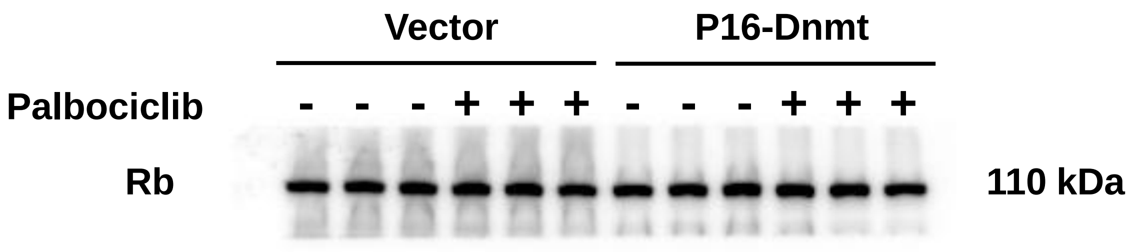

Figure 4d

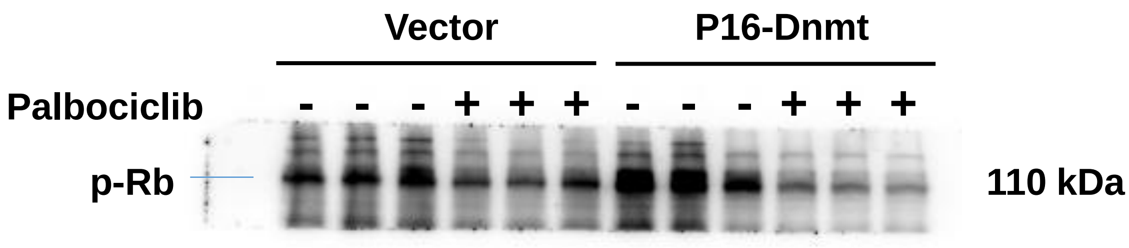

Figure 4d

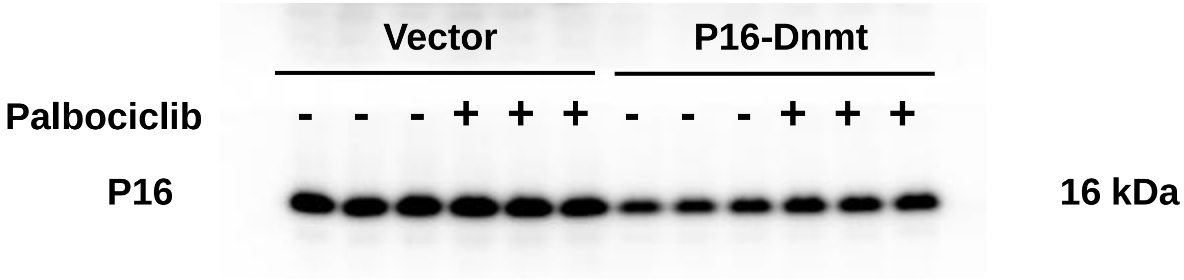

Figure 4d

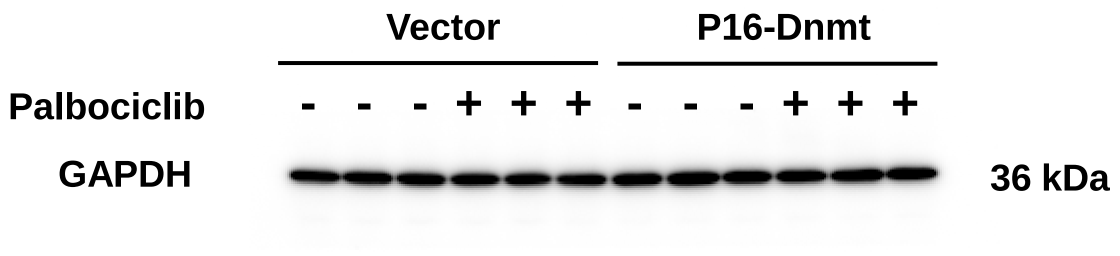

Supplement: S1 File — (PDF) [file pone.0223084.s003.pdf]
